# Assessing Hand Muscle Structural Modifications in Chronic Stroke

**DOI:** 10.3389/fneur.2018.00296

**Published:** 2018-05-08

**Authors:** Ya Zong, Henry H. Shin, Ying-Chih Wang, Sheng Li, Ping Zhou, Xiaoyan Li

**Affiliations:** ^1^Ruijin Hospital, School of Medicine, Shanghai Jiao Tong University, Shanghai, China; ^2^Guangdong Work Injury Rehabilitation Center, Guangzhou, China; ^3^Department of Physical Medicine and Rehabilitation, University of Texas Health Science Center at Houston, TIRR Memorial Hermann Research Center, Houston, TX, United States; ^4^Department of Occupational Science and Technology, University of Wisconsin-Milwaukee, Milwaukee, WI, United States

**Keywords:** stroke, electrical impedance myography, compound muscle action potential, hand, muscle

## Abstract

The purpose of the study is to assess poststroke muscle structural alterations by examining muscular electrical conductivity and inherent electrophysiological properties. In particular, muscle impedance and compound muscle action potentials (CMAP) were measured from the hypothenar muscle bilaterally using the electrical impedance myography and the electrophysiological techniques, respectively. Significant changes of muscle impedance were observed in the paretic muscle compared with the contralateral side (resistance: paretic: 27.54 ± 0.97 Ω, contralateral: 25.46 ± 0.91 Ω, *p* < 0.05; phase angle: paretic: 8.81 ± 0.61°, contralateral: 10.79 ± 0.69°, *p* < 0.05). In addition, impedance changes correlated moderately with the CMAP amplitude in the paretic hand (phase angle: *r* = 0.66, *p* < 0.05; reactance: *r* = 0.58, *p* < 0.05). The study discloses significant muscle rearrangements as a result of fiber loss or atrophy, fat infiltration or impaired membrane integrity in chronic stroke.

## Introduction

Muscle weakness is a remarkable symptom in stroke and contributes significantly to impaired motor functions. To understand mechanisms underlying weakness, studies can focus on assessing changes in neural control and muscular properties. In particular, intramuscular electromyography (EMG) and morphological techniques have been applied to examine muscle structural rearrangements poststroke. Increased motor unit fiber density, larger and complex motor unit action potentials (1–3), small angular fibers, as well as fiber type grouping (4, 5) have been observed in the acute and chronic stages of stroke suggesting the process of muscle denervation and reinnervation. While these studies characterize structural alterations in the paretic muscles, most approaches involve invasive recording and are limited by sampling only small selective areas of the muscle.

Electrical impedance myography (EIM) is an emerging technique for noninvasive evaluation of muscle electrical conductive properties. It applies weak, high-frequency alternating current to the muscles and produces raw bio-impedance data without causing neuronal and muscular depolarization (6, 7). EIM measures three impedance parameters in terms of resistance (*R*), reactance (*X*), and phase angle [θ = arctan (*X*/*R*)] (7, 8), which represent the inherent resistivity of skeletal muscle relative to extracellular and intracellular fluid, the integrity of cell membranes, tissue interfaces and non-ionic substances, and membrane oscillation properties of the muscle respectively (9–12).

Electrical impedance myography has been used to examine muscle structural alterations in a number of neuromuscular diseases including amyotrophic lateral sclerosis (ALS), muscular dystrophy, and spinal muscular atrophy (6, 7, 13–19). It is sensitive to muscle structural modifications in terms of atrophy, increased fat infiltration or connective tissue growth (20–22). In addition, the technique demonstrates strong correlations with standard measures of ALS including ALS functional rating scale-revised, handheld dynamometry, and motor unit number estimation in tracking the progression of the disease (13, 17, 23).

Applications of EIM to assess poststroke muscle conditions are relatively limited in the literature. In a previous study, we examined muscle impedance properties in the biceps brachii and found significant changes of muscle structural properties in the paretic side (24). Since proximal muscles demonstrate different extents of impairment from distal muscles (25), it remains unknown whether findings from biceps brachii are applicable to hand muscles. In this study, we applied EIM technique to examine impedance changes in the hypothenar muscle poststroke. In addition, we measured the compound muscle action potentials (CMAP) of the muscle, to assess inherent electrical properties. CMAP is evoked by electrical activation of all functioning motor units and represents summation of all action potentials in spatial distribution. Application of the two different techniques to the same muscle may disclose different features of the muscle and improve current knowledge on structural changes in the paretic hand muscle.

## Materials and Methods

### Experiment

#### Subjects

Fourteen chronic stroke survivors participated in the study (8 female, 6 male, age: 63 ± 10 years, mean ± SD). They had a single incidence of stroke with the time course of stroke varying from 8 month to 15 years (80 ± 55 months, mean ± SD). All subjects were free of any other known neurological disorders or symptoms including neuropathy, radiculopathy, cervical spondylosis, or hyperglycemia. All subjects gave written informed consent in accordance with the Declaration of Helsinki. The protocol was approved by the Protection of Human Subjects (CPHS) at University of Texas Health Science Center at Houston.

Clinical assessment: hand recovery was evaluated using grip force and the Chedoke–McMaster assessment.

#### Experiment

Experimental protocol included EIM and electrophysiological tests in the hypothenar muscle.

##### EIM Measurement

Subjects were seated upright with the examined arm in a natural, resting position on a height-adjustable table. Muscle impedance was measured using the mView EIM system (Myolex Inc., Boston, MA, USA). A handheld electrode array (P/N number: 20-00036) was placed on the muscle bulk with slight pressure applied by the experimenter. To further improve contact between the electrode and skin, saline wipe was used to moisten the skin before each trial. During the trial, alternating current at high frequencies from 1 kHz to 10 MHz was delivered to the muscle in discrete steps. Stimulation intensity was less than 1 mA which did not involve any neuronal or muscular depolarization. Impedance measurement was applied to each muscle multiple times until three consistent trials were saved. In this study, impedance was obtained from the pair of current electrodes with the largest inter-electrode distance of 32 mm in parallel to muscle fiber direction.

##### Muscle Response Measurement

Subsequent to the EIM test, CMAP was collected from the hypothenar muscle using an UltraPro S100 EMG system (Natus Neurology Incorporated, Middleton, WI, USA). Two disposable electrodes (Ag–AgCl electrode, 10-mm diameter) were placed on the motor point of the muscle and the distal phalanx of the little finger, respectively, as the active and reference electrodes. The ground electrode was positioned on the dorsal side of the hand. A standard bar electrode was placed on the ulnar nerve, 2 cm proximal to the wrist crease with the cathode oriented distally. After the electrodes were securely attached, the examined hand was restrained in pronation by Nylatex^®^ wraps (4″ width).

Electrical stimulation was applied in single impulses of 200 µs width. It was initiated from relatively low intensity and increased at 2 mA per step until the maximum muscle response was reached. To guarantee all motor units were activated, supramaximal stimulation was applied to record the CMAP. Muscle responses were sampled at a frequency of 48 kHz and band-pass filtered between 1 Hz and 10 kHz.

### Data Analysis

Electrical impedance myography and muscle response data were processed using the Matlab software (MathWorks, Natick, MA, USA).

#### EIM Analysis

Impedance variables including resistance (*R*), reactance (*X*), and phase angle (θ) were averaged across three trials at the frequency of 100 kHz for comparison.

#### Muscle Responses

Compound muscle action potential amplitude was measured as the difference between negative peak and baseline of the waveform.

All data were screened for outliers and normality of distribution. Paired *t*-test was applied to compare the differences of impedance variables (*R, X*, and θ) and muscle responses (CMAP amplitude) between paretic and contralateral muscles. Pearson correlation analysis was used to assess linear relationships between EIM variables and the CMAP amplitude. Clinical relevance was examined by calculating Spearman ρ coefficients between impedance variables or muscle responses and the Chedoke scores. Pearson coefficients were calculated between two clinical measures of grip force and duration of the stroke, and the EIM or CMAP amplitude. Statistical significance was set as *p* < 0.05. Results are reported in a mean ± SE format unless specified.

## Results

**Figure 1:**
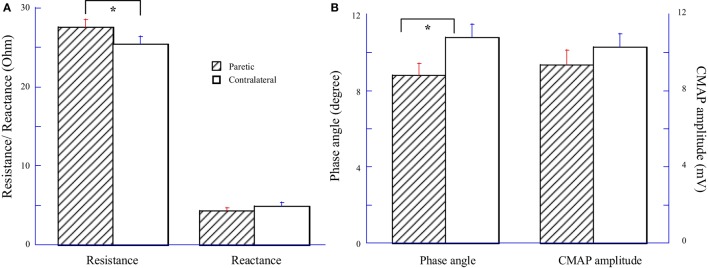
Comparisons of electrical impedance myography and compound muscle action potential (CMAP) between paretic and contralateral muscles. **(A)** Resistance (R) and reactance (X). **(B)** Phase angle (θ) and CMAP amplitude.

Impedance variables and CMAP amplitude were averaged across 14 subjects and compared between the paretic and contralateral muscles as illustrated in Figure 1. Among them, eight subjects had paresis in the dominant hand and six subjects had paresis in the non-dominant side. Statistical analysis indicated a significant increase of resistance (*R*) and a significant decrease of phase angle (θ) in the paretic hypothenar muscle compared with the contralateral side (*R*: paretic: 27.54 ± 0.97 Ω, contralateral: 25.46 ± 0.91 Ω, *p* < 0.05; θ: paretic: 8.81 ± 0.61°, 10.79 ± 0.69°, *p* < 0.05). On the other hand, no significant differences in the reactance (*X*) and the CMAP amplitude were observed between two sides (*X*: paretic: 4.32 ± 0.37 Ω, contralateral: 4.91 ± 0.43 Ω, *p* = 0.19; CMAP: paretic: 9.34 ± 0.79 mV, contralateral: 10.28 ± 0.7 mV, *p* = 0.19).

Significant weakness was observed in the paretic side from measurement of grip force (paretic: 7.84 ± 1.68 kg, contralateral: 29.44 ± 1.91 kg, *p* < 0.001). Chedoke test disclosed severe impairment of hand functions in most subjects (score 1:3 subjects, score 2:4 subjects, and score 3:5 subjects) except two subjects who had scores of 6 and 7, respectively. Pearson correlation coefficients between the EIM variables (*R, X*, and θ) and the CMAP amplitude were illustrated in Table 1. In particular, moderate correlations between the CMAP amplitude and the phase angle (θ) were observed in both the paretic and contralateral muscles (paretic: *r* = 0.66, *p* < 0.05; contralateral: *r* = 0.53, *p* < 0.05). The CMAP amplitude also correlated moderately with the reactance (*X*) in the paretic muscle (*r* = 0.58, *p* < 0.05). We did not find any other significant correlations between impedance and CMAP amplitude on either side. In addition, changes of impedance or CMAP amplitude were not associated with any clinical measures including grip force, Chedoke assessments, and the time course of stroke (*p* > 0.1).

**Table 1:** Pearson correlation coefficients between electrical impedance myography and compound muscle action potential (CMAP).

	p_CMAP	c_CMAP	p_R	c_R	p_X	c_X	p_θ	c_θ
p_CMAP	1.00	0.58*	0.01	−0.12	0.58*	0.17	0.66*	0.28
c_CMAP		1.00	−0.01	0.00	0.38	0.39	0.42	0.53*
p_R			1.00	0.54*	0.58*	0.24	0.26	0.07
c_R				1.00	0.24	0.68**	0.03	0.33
p_X					1.00	0.42	0.94**	0.43
c_X						1.00	0.38	0.91**
p_θ							1.00	0.48
c_θ								1.00

*p, paretic; c, contralateral*.

Significance was marked in bold *p < 0.05; **p < 0.01.

## Discussion

The present study assessed structural alterations in the hypothenar muscle in chronic stroke using EIM and electrophysiological techniques. In particular, significant changes of impedance variables in terms of increased resistance and decreased phase angle were observed in the paretic muscle compared with the contralateral side. Additionally, changes of muscle impedance correlated well with the CMAP amplitude in the paretic side.

### Impedance Changes

This study confirms our previous findings of impedance change in stroke that both the distal and proximal muscles of the upper limb demonstrated substantial reduction in phase angle in the paretic side (24). Phase angle is a sensitive biomarker not only for evaluation of muscle state in neurological disorders but also for assessment of malnutrition, cancer, and heart failure (9, 13, 26, 27). It derives from the ratio of the resistance and reactance and represents membrane oscillation regardless of the anatomical differences. Despite that the physiological meaning and pathologic effects are not fully understood (28), a lower phase angle is often associated with muscle loss and decreased cell integrity (9, 29).

Compared with our previous study that observed remarkable reduction of reactance in the biceps muscle (24), this study exhibited significant changes of resistance in the hypothenar muscle following a stroke. A number of factors may account for the different results of the two studies, including subject group, electrode array size, subcutaneous fat thickness, and the frequency used for analysis (30, 31). The differences are also likely due to that the proximal and distal muscles can be differently affected after stroke.

Phase angle and resistance are reported to have strong correlations with subcutaneous fat thickness (31). With stroke and decreased mobility, there is a tendency of increase of fat mass in the paretic muscle replacing the lost muscle mass (32). Changes of phase angle and resistance in the study may reflect muscle structural reorganization in terms of increased fat thickness and/or reduced muscle mass in the paretic muscle. On the other hand, the data were obtained from eight subjects with paresis in the dominant hand and six subjects with paresis in the non-dominant hand. It seems that handedness has limited influence on changes of impedance in the paretic muscle.

### Correlations Between EIM and CMAP

Phase angle and CMAP quantify two different electrical properties of the muscle. In general, CMAP amplitude measures muscle response in the forms of depolarization and repolarization whereas EIM measures muscle electrical conductivity as the low-intensity current passes the tissue. Despite the differences, the two parameters can quantify loss of muscle mass and correlate moderately in the paretic side in this study.

Decrease of CMAP amplitude in the paretic side was observed in the study, though statistical analysis did not show any significant difference between the paretic and contralateral sides. The results are slightly different from previous studies in stroke (33, 34). In the chronic stroke, CMAP amplitude can be partially compensated by collateral sprouting of the denervated muscle fibers (35, 36). As a result, CMAP amplitude may maintain in a low normal range or be slightly lower than normal. Nevertheless, current findings may indicate high sensitivity of the EIM technique in detection of muscle structural alterations when electrophysiological measures may not disclose any significant changes.

### Clinical Relevance

This study did not find any significant correlations between clinical assessments (grip force, time course of stroke, and Chedoke score) and EIM or CMAP measures. Grip force and Chedoke evaluation involve coordination of multiple intrinsic and extrinsic hand muscles, for which hypothenar muscle has very limited contributions. This may explain the insignificant correlations in the study. In addition, Chedoke assessment uses a 7-point ordinal scale to differentiate levels of hand function recovery. The subjective nature of the assessment and tendency to cluster in the lower range in the current study may also lead to insignificant relationship. In our previous study, we also observed that changes of EIM are not associated with the time course of stroke in the biceps (24). It seems state of the paretic muscle remains relatively stable in the chronic stage of stroke. Future work involving direct measurement of maximal contraction force from the tested muscle or using multiple muscles may disclose new information on the relation of EIM and clinical assessments.

### Limitations

There are a number of limitations in the study. To match the muscle size, this study used the electrode array with relative smaller inter-electrode distance compared with previous studies (37, 38). When current electrodes are closer to the voltage electrodes, a larger proportion of the current will pass through the subcutaneous layer rather than deeper through the muscle. As a result, the measurement reflects a mix data of muscle and fat which undermines the real impedance measured from muscle (31). Since EIM (reactance and phase angle) recorded from the small electrode demonstrate high percent of negative values at 50 kHz, current results were based on impedance at 100 kHz. As suggested in the literature, healthy tissue is more reactive around 50 kHz, whereas tissues with disease may attain a peak reactance at higher frequencies (20). Use of impedance at 100 kHz may limit the comparison of the findings to other studies at 50 kHz.

## Conclusion

This study found significant changes of impedance in the paretic hypothenar muscles compared with the contralateral side. The correlations between EIM and electrophysiological measurement may indicate high sensitivity of the EIM technique in detection of muscle structural alterations. Future studies involving multiple frequency analysis may reveal insights for better understanding the impedance changes associated with structural modifications.

## Ethics Statement

The study was approved by Institutional Review Board of University of Texas Health Science Center and the TIRR Memorial Herman on 07/16/2016 (IRB #: HSC-MS-16-0197).

## Author Contributions

YZ performed data analysis and interpretation, helped data collection, and wrote the first draft of the manuscript. HS performed data collection and helped data analysis. Y-CW participated in experiment design, helped data analysis and interpretation. SL participated in experiment design, provided clinical support for the study, and helped data analysis and interpretation. PZ participated in experiment design, helped data analysis and interpretation, and oversaw the study. XL performed experiment design, helped data collection, analysis, and interpretation, and critically revised the manuscript. All the authors read, revised, and approved the final manuscript.

## Conflict of Interest Statement

The authors declare that the research was conducted in the absence of any commercial or financial relationships that could be construed as a potential conflict of interest.

## Abbreviations

EIM, electrical impedance myography; CMAP, compound muscle action potential.

## References

[1] Cruz Martinez A Ferrer MT Morales C Perez Conde MC Calatayud T Mingo P Electromyography, single fibre electromyography and necropsy findings in myasthenic syndrome associated with bronchogenic carcinoma. Electromyogr Clin Neurophysiol (1982) 22(7):531–48. 6295740

[2] Lukacs M Vecsei L Beniczky S. Changes in muscle fiber density following a stroke. Clin Neurophysiol (2009) 120(8):1539–42. 10.1016/j.clinph.2009.06.001 19564129

[3] Lukacs M. Electrophysiological signs of changes in motor units after ischaemic stroke. Clin Neurophysiol (2005) 116(7):1566–70. 10.1016/j.clinph.2005.04.005 15905127

[4] Dattola R Girlanda P Vita G Santoro M Roberto ML Toscano A Muscle rearrangement in patients with hemiparesis after stroke: an electrophysiological and morphological study. Eur Neurol (1993) 33(2):109–14. 10.1159/000116915 8467816

[5] Segura RP Sahgal V Hemiplegic atrophy: electrophysiological and morphological studies. Muscle Nerve (1981) 4(3):246–8. 10.1002/mus.880040312 7242561

[6] Esper GJ Shiffman CA Aaron R Lee KS Rutkove SB. Assessing neuromuscular disease with multifrequency electrical impedance myography. Muscle Nerve (2006) 34(5):595–602. 10.1002/mus.20626 16881067

[7] Rutkove SB Aaron R Shiffman CA. Localized bioimpedance analysis in the evaluation of neuromuscular disease. Muscle Nerve (2002) 25(3):390–7. 10.1002/mus.10048 11870716

[8] Kyle UG Bosaeus I De Lorenzo AD Deurenberg P Elia M Gomez JM Bioelectrical impedance analysis – part I: review of principles and methods. Clin Nutr (2004) 23(5):1226–43. 10.1016/j.clnu.2004.06.004 15380917

[9] Mulasi U Kuchnia AJ Cole AJ Earthman CP. Bioimpedance at the bedside: current applications, limitations, and opportunities. Nutr Clin Pract (2015) 30(2):180–93. 10.1177/0884533614568155 25613832

[10] Lukaski HC. Evolution of bioimpedance: a circuitous journey from estimation of physiological function to assessment of body composition and a return to clinical research. Eur J Clin Nutr (2013) 67(Suppl 1):S2–9. 10.1038/ejcn.2012.149 23299867

[11] Matthie JR. Bioimpedance measurements of human body composition: critical analysis and outlook. Expert Rev Med Devices (2008) 5(2):239–61. 10.1586/17434440.5.2.239 18331184

[12] Ellis KJ Bell SJ Chertow GM Chumlea WC Knox TA Kotler DP Bioelectrical impedance methods in clinical research: a follow-up to the NIH Technology Assessment Conference. Nutrition (1999) 15(11–12):874–80. 10.1016/S0899-9007(99)00147-1 10575664

[13] Rutkove SB Caress JB Cartwright MS Burns TM Warder J David WS Electrical impedance myography as a biomarker to assess ALS progression. Amyotroph Lateral Scler (2012) 13(5):439–45. 10.3109/17482968.2012.688837 22670883 PMC3422377

[14] Garmirian LP Chin AB Rutkove SB. Discriminating neurogenic from myopathic disease via measurement of muscle anisotropy. Muscle Nerve (2009) 39(1):16–24. 10.1002/mus.21115 19058193 PMC2719295

[15] Rutkove SB Geisbush TR Mijailovic A Shklyar I Pasternak A Visyak N Cross-sectional evaluation of electrical impedance myography and quantitative ultrasound for the assessment of Duchenne muscular dystrophy in a clinical trial setting. Pediatr Neurol (2014) 51(1):88–92. 10.1016/j.pediatrneurol.2014.02.015 24814059 PMC4063877

[16] Arnold W McGovern VL Sanchez B Li J Corlett KM Kolb SJ The neuromuscular impact of symptomatic SMN restoration in a mouse model of spinal muscular atrophy. Neurobiol Dis (2016) 87:116–23. 10.1016/j.nbd.2015.12.014 26733414 PMC4724465

[17] Rutkove SB Shefner JM Gregas M Butler H Caracciolo J Lin C Characterizing spinal muscular atrophy with electrical impedance myography. Muscle Nerve (2010) 42(6):915–21. 10.1002/mus.21784 21104866

[18] Nichols C Jain MS Meilleur KG Wu T Collins J Waite MR Electrical impedance myography in individuals with collagen 6 and laminin α-2 congenital muscular dystrophy: a cross-sectional and 2-year analysis. Muscle Nerve (2018) 57(1):54–60. 10.1002/mus.25629 28224647 PMC6383203

[19] Li Z Tian D Chen L Wang X Jiang L Yu Y. Electrical impedance myography for discriminating traumatic peripheral nerve injury in the upper extremity. Clin Neurophysiol (2017) 128(2):384–90. 10.1016/j.clinph.2016.11.016 27940046

[20] Rutkove SB. Electrical impedance myography: background, current state, and future directions. Muscle Nerve (2009) 40(6):936–46. 10.1002/mus.21362 19768754 PMC2824130

[21] Li J Geisbush TR Rosen GD Lachey J Mulivor A Rutkove SB. Electrical impedance myography for the *in vivo* and *ex vivo* assessment of muscular dystrophy (mdx) mouse muscle. Muscle Nerve (2014) 49(6):829–35. 10.1002/mus.24086 24752469 PMC5582805

[22] Li Z Chen L Zhu Y Wei Q Liu W Tian D Handheld electrical impedance myography probe for assessing carpal tunnel syndrome. Ann Biomed Eng (2017) 45(6):1572–80. 10.1007/s10439-017-1819-3 28361183

[23] Rutkove SB Caress JB Cartwright MS Burns TM Warder J David WS Electrical impedance myography correlates with standard measures of ALS severity. Muscle Nerve (2014) 49(3):441–3. 10.1002/mus.24128 24273034

[24] Li X Li L Shin H Li S Zhou P. Electrical impedance myography for evaluating paretic muscle changes after stroke. IEEE Trans Neural Syst Rehabil Eng (2017) 25(11):2113–21. 10.1109/TNSRE.2017.2707403 28574361 PMC6734926

[25] Twitchell TE The restoration of motor function following hemiplegia in man. Brain (1951) 74(4):443–80. 10.1093/brain/74.4.443 14895765

[26] Gupta D Lammersfeld CA Vashi PG King J Dahlk SL Grutsch JF Bioelectrical impedance phase angle as a prognostic indicator in breast cancer. BMC Cancer (2008) 8:249. 10.1186/1471-2407-8-249 18727837 PMC2527613

[27] Colin-Ramirez E Castillo-Martinez L Orea-Tejeda A Vazquez-Duran M Rodriguez AE Keirns-Davis C. Bioelectrical impedance phase angle as a prognostic marker in chronic heart failure. Nutrition (2012) 28(9):901–5. 10.1016/j.nut.2011.11.033 22465907

[28] Barbosa-Silva MC Barros AJ Wang J Heymsfield SB Pierson RN Jr. Bioelectrical impedance analysis: population reference values for phase angle by age and sex. Am J Clin Nutr (2005) 82(1):49–52. 10.1093/ajcn/82.1.49 16002799

[29] Rutkove SB Esper GJ Lee KS Aaron R Shiffman CA. Electrical impedance myography in the detection of radiculopathy. Muscle Nerve (2005) 32(3):335–41. 10.1002/mus.20377 15948202

[30] Kortman HG Wilder SC Geisbush TR Narayanaswami P Rutkove SB. Age- and gender-associated differences in electrical impedance values of skeletal muscle. Physiol Meas (2013) 34(12):1611–22. 10.1088/0967-3334/34/12/1611 24165434 PMC3895401

[31] Sung M Spieker AJ Narayanaswami P Rutkove SB. The effect of subcutaneous fat on electrical impedance myography when using a handheld electrode array: the case for measuring reactance. Clin Neurophysiol (2013) 124(2):400–4. 10.1016/j.clinph.2012.07.013 22917581 PMC3543755

[32] English C McLennan H Thoirs K Coates A Bernhardt J. Loss of skeletal muscle mass after stroke: a systematic review. Int J Stroke (2010) 5(5):395–402. 10.1111/j.1747-4949.2010.00467.x 20854624

[33] Arasaki K Igarashi O Ichikawa Y Machida T Shirozu I Hyodo A Reduction in the motor unit number estimate (MUNE) after cerebral infarction. J Neurol Sci (2006) 250(1–2):27–32. 10.1016/j.jns.2006.06.024 16904126

[34] Li X Fisher M Rymer WZ Zhou P. Application of the F-response for estimating motor unit number and amplitude distribution in hand muscles of stroke survivors. IEEE Trans Neural Syst Rehabil Eng (2016) 24(6):674–81. 10.1109/TNSRE.2015.2453274 26168437 PMC4902775

[35] Hara Y Akaboshi K Masakado Y Chino N. Physiologic decrease of single thenar motor units in the F-response in stroke patients. Arch Phys Med Rehabil (2000) 81(4):418–23. 10.1053/mr.2000.3872 10768529

[36] Li X Suresh A Zhou P Rymer WZ. Alterations in the peak amplitude distribution of the surface electromyogram poststroke. IEEE Trans Biomed Eng (2013) 60(3):845–52. 10.1109/TBME.2012.2205249 22736632

[37] Narayanaswami P Spieker AJ Mongiovi P Keel JC Muzin SC Rutkove SB. Utilizing a handheld electrode array for localized muscle impedance measurements. Muscle Nerve (2012) 46(2):257–63. 10.1002/mus.23307 22806375 PMC3400114

[38] Li X Shin H Li L Magat E Li S Zhou P. Assessing the immediate impact of botulinum toxin injection on impedance of spastic muscle. Med Eng Phys (2017) 43:97–102. 10.1016/j.medengphy.2017.01.018 28169197 PMC5382061

